# The components data of fuzheng huayu extracts, *cordyceps sinensis mycelia* polysaccharide, gypenosides and amygdalin

**DOI:** 10.1016/j.dib.2019.104087

**Published:** 2019-06-01

**Authors:** Wei Liu, Zhixiong Li, Zhaolin Sun, Yongbin Xu, Shunchun Wang, Yiyang Hu, Jinghua Peng

**Affiliations:** a Institute of Liver Diseases, Shuguang Hospital Affiliated to Shanghai University of Traditional Chinese Medicine, Shanghai, 201203, China; b Institute of Clinical Pharmacology, Shuguang Hospital Affiliated to Shanghai University of Traditional Chinese Medicine, Shanghai, 201203, China; c Shanghai Institute of Materia Medica, Chinese Academy of Sciences, Shanghai 201203, China; d Key Laboratory of Liver and Kidney Diseases (Shanghai University of Traditional Chinese Medicine), Ministry of Education, Shanghai, 201203, China; e Shanghai Key Laboratory of Traditional Chinese Clinical Medicine, Shanghai, 201203, China; f Institute of Chinese Materia Medica, Shanghai University of Traditional Chinese Medicine, Shanghai, 201203, China

**Keywords:** High-performance liquid chromatography, Traditional Chinese medicine, Fuzheng huayu capsule, Cordyeps sinensis polysaccharide, Gypenosides

## Abstract

Fuzheng Huayu (FZHY) capsule is a traditional Chinese medicine composed of six Chinese medicinal herbs Tian et al. [1] and approved by China food and drug administration for liver fibrosis treatment [2], [3] Liu et al., 2009 and Liu et al., 2005. CGA formula consisting of Cordyeps sinensis polysaccharide (CS-PS), gypenosides (G), and amygdalin (A), are derived from FZHY formula. It is necessary to identify the chemical profile of FZHY and CGA formula to describe the mechanisms and the corresponding components of anti-fibrosis. It is showed that FZHY contains adenosine (5.21 mg/g), amygdalin (5.31 mg/g), salvianolic acid b (18.22 mg/g) and deoxyschizandrin (2.62 mg/g), respectively. CS-PS contained 60.5 ± 2.2% total carbohydrate, including 14.17% arabinose, 25.35% glucose and 60.48% galactose. Gypenosides contain 10.34% gypenosides XLIX and 16.58% gypenosides A. These data provide the primary chemical profile of FZHY and CGA formula and an example for components analysis of traditional Chinese medicine.

**Table:** Specifications table

Subject area	*Chemistry*
More specific subject area	*Chemical analysis of phytomedicine*
Type of data	*Tables and figures*
How data was acquired	*Gypenosides was analyzed with high-pressure liquid chromatography (Agilent 1200 Series, USA). Total carbohydrate content of CS-PS was determined by the phenol-sulphuric acid method. The carbohydrate composition of CS-PS was analyzed by gas chromatograph-mass spectrometer (Thermo Fisher Scientific Inc., FL, USA). The components of FZHY extract was analyzed by using ultra-high-performance liquid chromatography-Q exactive hybrid quadrupole orbitrap high-resolution accurate mass spectrometric (UHPLC-Q-Orbitrap HRMS, Thermo Fisher Scientific Inc., Grand Island, NY, USA).*
Data format	*Raw*
Experimental factors	*Before mass spectroscopy,* FZHY extracts and gypenosides were dissolved by methanol. CS-PS was hydrolyzed by trifluoroacetic acid, then was vacuum dried and dissolved in methanol.
Experimental features	*Samples were prepared and analyzed according to the protocol described previously*[1], [4], [5]*.*
Data source location	*Shanghai, China*
Data accessibility	*Data is with this article*
Related research article	*Huajie Tian, Lin Liu, Zhixiong Li, Wei Liu, Zhaolin Sun, Yongbin Xu, Shunchun Wang, Chungeng Liang, Yamei Hai, Qin Feng, Yu Zhao, Yiyang Hu, Jinghua Peng. Chinese medicine CGA formula ameliorates liver fibrosis induced by carbon tetrachloride involving inhibition of hepatic apoptosis in rats. Journal of ethnopharmacology 2019; 232: 227–35. DOI:**10.1016/j.jep.2018.11.027*[*1*]*.*

**Table:** 

**Value of the data** • People can find and cite the components of Fuzheng Huayu (FZHY) capsule which is approved by China food and drug administration for treatment of liver fibrosis. • The data provides reference of the carbohydrate content and components of Cordyeps sinensis polysaccharide (CS-PS). • People can find and reference the components of Herba Gynostemmae gypenosides. • People can find and cite the chemical components of CGA formula, which is derived from FZHY and has been reported in several research articles.

## 1 Data

**Fig. 1:**
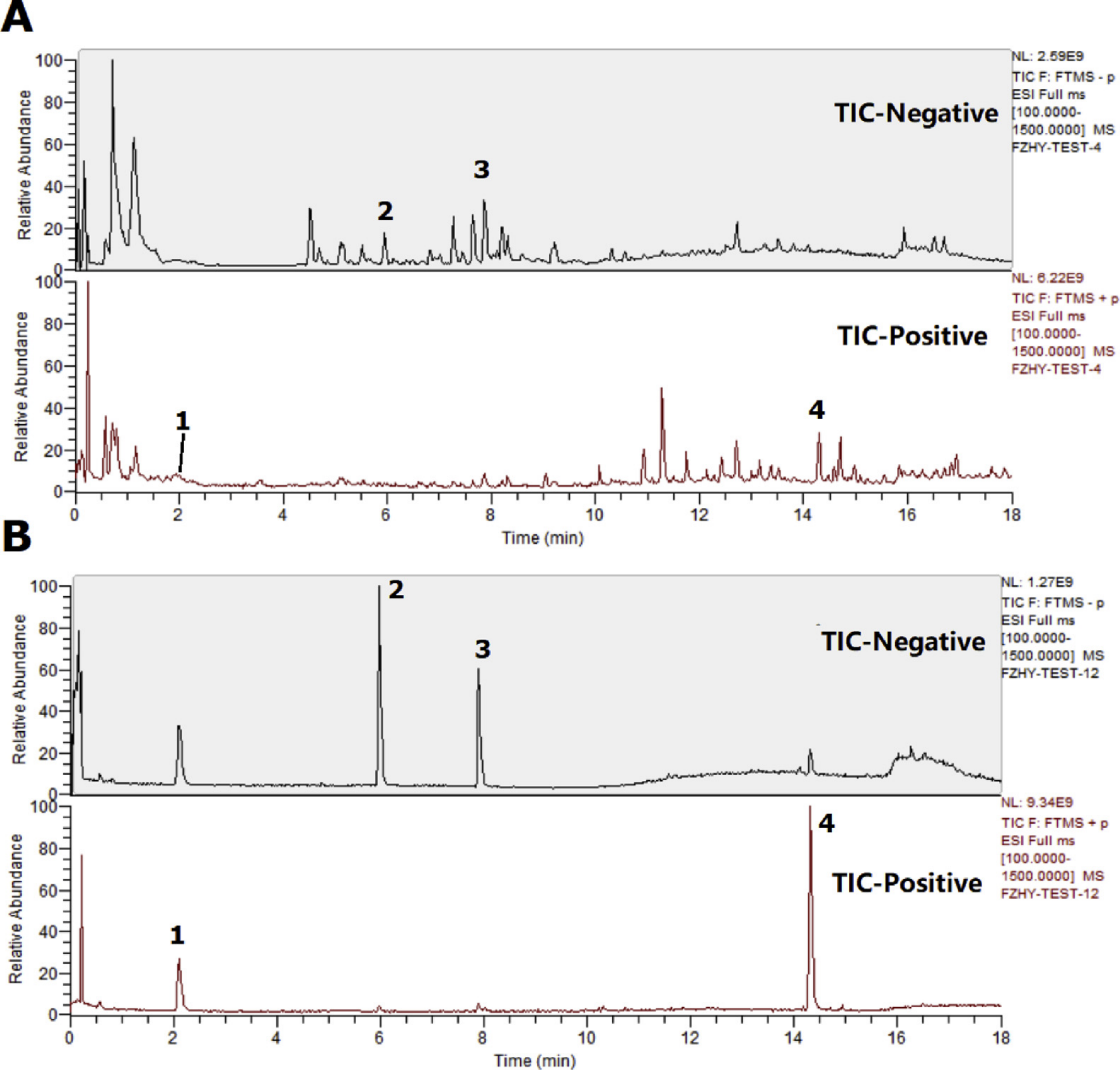
**The typical LC-MS fingerprint chromatograms of extract of FZHY (A) and mixture reference standards (B).** TIC, Total-ion chromatograms, 1, Adenosine, 2, amygdalin, 3, salvianolic acid b, 4, deoxyschizandrin. The contents of adenosine, amygdalin, salvianolic acid b and deoxyschizandrin in FZHY extract were determined as 5.21 mg/g, 5.31 mg/g, 18.22 mg/g and 2.62 mg/g, respectively.

**Fig. 2:**
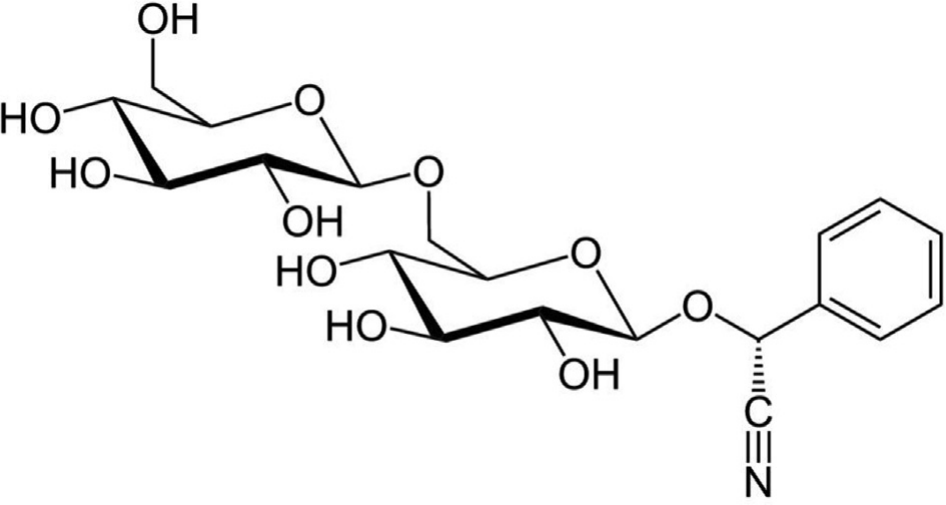
**Chemical structure of Amygdalin**.

**Fig. 3:**
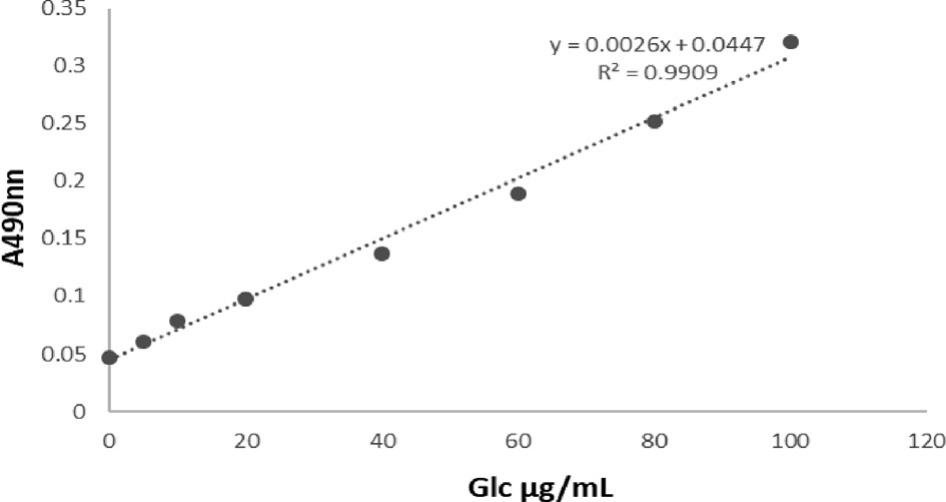
**Standard curve of total carbohydrate content determined by phenol-sulfuric acid method.** A490 nm, the absorbance of standard samples on 490 nm, Glc μg/ml, the content of glucose.

**Fig. 4:**
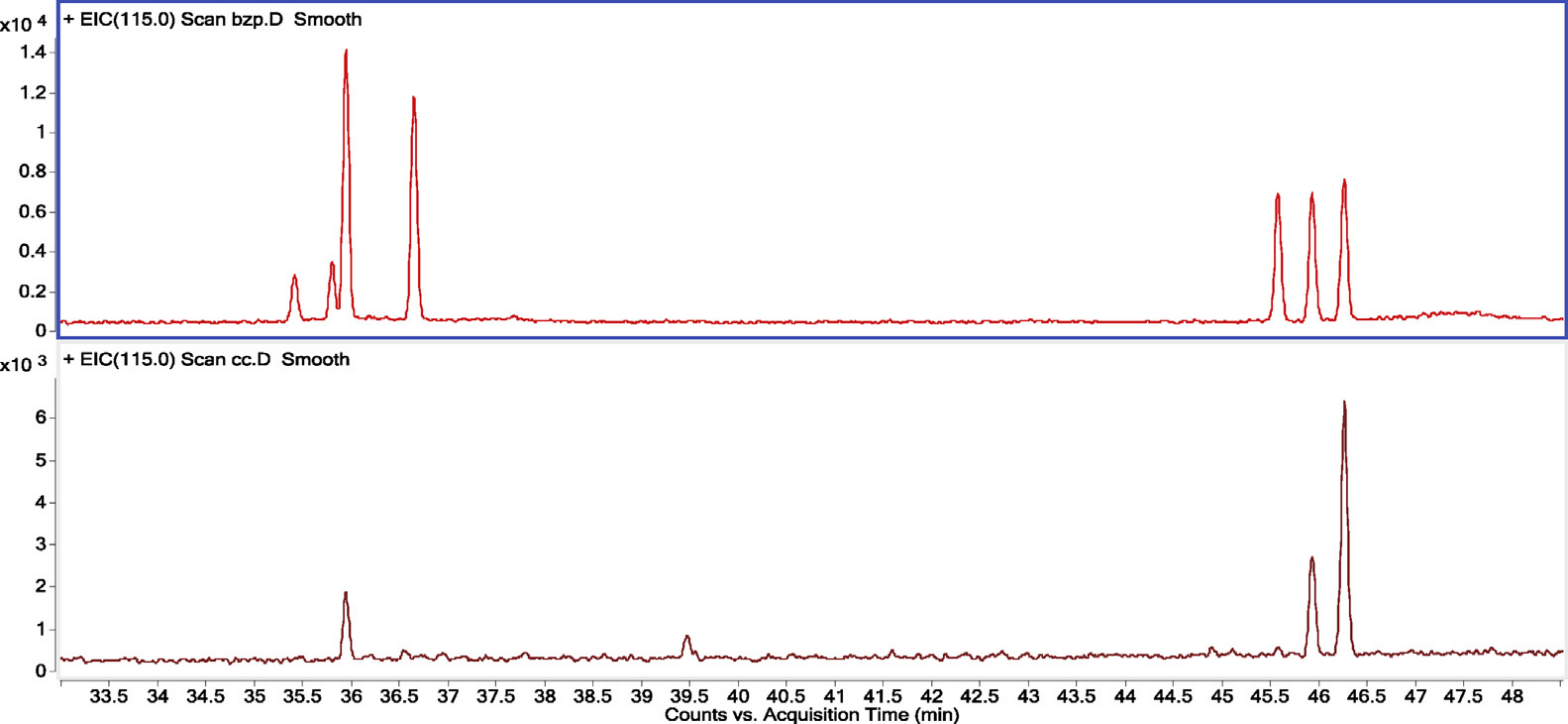
**Chemical components of CS-PS (**bottom image**) and standard (**above image, ribose, arabinose, xylose, mannose, glucose, galactose**) analyzed by gas chromatograph-mass spectrometer (GC-MS) (TRACE-DSQ, Thermo Fisher Scientific Inc., FL, USA).** Above image, standard analysis showed that CS-PS contained 14.17% arabinose, 25.35% glucose and 60.48% galactose.

**Fig. 5:**
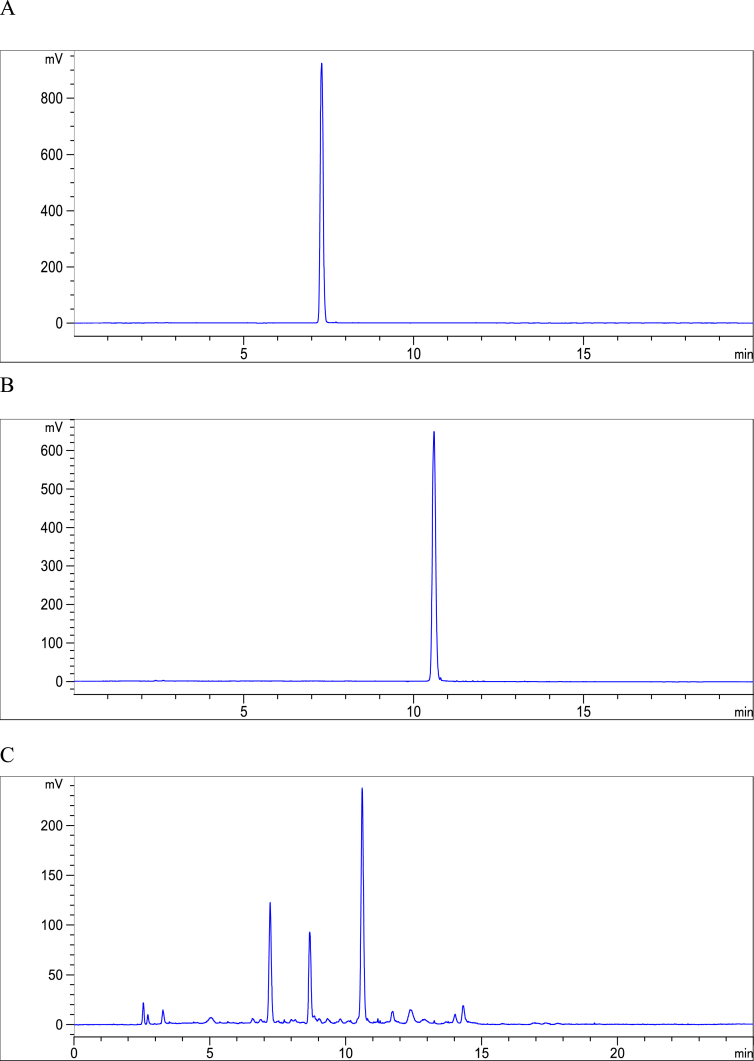
**HPLC chromatograms of gypenosides and the standard compounds.** (A), gypenoside XLIX, (B), gypenosides A, (C), gypenosides Gypenosides contained 10.34% gypenosides XLIX and 16.58% gypenosides A.

The data presented here provided the primary chemical profile of FZHY (formula shown in Table 1) and CGA formula. In FZHY, the contents of adenosine, amygdalin, salvianolic acid b and deoxyschizandrin extracts were determined as 5.21 mg/g, 5.31 mg/g, 18.22 mg/g and 2.62 mg/g respectively (Fig. 1). In CGA formula, the molecular structure of amygdalin (Fig. 2) and the chemical profile of cordyeps sinensis polysaccharide and gypenosides were determined. The total carbohydrate content of cordyeps sinensis polysaccharide was determined as 60.5 ± 2.2% (Fig. 3). The carbohydrate composition analysis showed cordyeps sinensis polysaccharide contained 14.17% arabinose, 25.35% glucose and 60.48% galactose (Fig. 4). Gypenosides contained 10.34% gypenosides XLIX and 16.58% gypenosides A (Fig. 5).

**Table 1:** The formula of FZHY.

Pharmaceutical name	Botanical name	Family and plant part use	Chinese name	% (w/w)
Salviae Miltiorrhizae Radix et Rhizoma	*Salvia miltiorrhiza* Bunge	*Lamiaceae;* radix;	Danshen	33.3
Artificial fermentation cordyceps	*Cordyceps sinensis* (BerK.) Sacc.	*Clavicipitaceae*; mycelia	Chongcao	16.7
Persicae Semen	*Prunus persica* (L.) Batsch	*Rosaceae;* seed	Taoren	8.3
*Herba Gynostemmae	*Gynostemma pentaphyllum* (Thunb.) Makino	*Cucurbitaceae;* whole herb	Jiaogulan	25
Pini Pollen	*Pinus massoniana* Lamb.	*Pinaceae;* pollen	Songhuafen	8.3
Schisandrae Chinensis Fructus	*Schisandra chinensis* (Turcz.) Baill.	*Schisandraceae;* fruit	Wuweizi	8.3

*The botanical name of Jiaogulan is from The Drug Standard of Gugangxi Province (1996 Edition), the others are from The Pharmacopoeia of the People's Republic of China (2015 Edition). The botanical names have been updated with www.theplantlist.org.

## 2 Experimental design, materials and methods

### 2.1 Materials

The formula of FZHY [2], [3] was presented in Table 1. The FZHY extracts (batch number: 180206) were prepared and provided by Shanghai Sundise Medicine Technology Development Co., Ltd. (Shanghai, China). *Cordyeps sinensis* polysaccharide (CS-PS, purity 62.6%), Herba Gynostemmae gypenosides (purity 92.8%) and amygdalin ([(6-O-β-D-glucopyranosyl-β-D-glucopyranosyl)oxy](phenyl)acetonitrile) (Fig. 2, purity 98%) were prepared and provided by Shanghai Institute of Materia Medica, Chinese Academy of Sciences (Shanghai, China).

### 2.2 Methods

FZHY extracts were analyzed by ultra-high-performance liquid chromatography-Q exactive hybrid quadrupole orbitrap high-resolution accurate mass spectrometric (UHPLC-Q-Orbitrap HRMS, Thermo Fisher Scientific Inc., Grand Island, NY, USA) [1] and standards of the components in FZHY identified previously [6], [7], [8], [9]. FZHY extracts contain adenosine (5.21 mg/g), amygdalin (5.31 mg/g), salvianolic acid b (18.22 mg/g) and deoxyschizandrin (2.62 mg/g) (Fig. 1).

Phenol-sulfuric acid method (Table 2) was employed to determin the total carbohydrate content of CS-PS [4], [5]. The total carbohydrate content of CS-PS was 60.5 ± 2.2% based on the calibration curve (Fig. 3). The carbohydrate composition of CS-PS was analyzed by gas chromatograph-mass spectrometer (GC-MS) (Thermo Fisher Scientific Inc, Waltham, MA, USA) [4], [5], including 14.17% arabinose, 25.35% glucose and 60.48% galactose (Fig. 4).

**Table 2:** The procedure of reagents admixture in phenol-sulfuric acid method.

Reagents	Standard 1 (ml)	Standard 2 (ml)	Standard 3 (ml)	Standard 4 (ml)	Standard 5 (ml)	Standard 6 (ml)	Standard 7 (ml)	CS-PS sample (ml)
48μg/ml glucose	0	0.4	0.7	1.0	1.3	1.6	2	0
deionized water	2	1.6	1.3	1.0	0.7	0.4	0	0
CS-PS sample solution	0	0	0	0	0	0	0	2
6% phenol	1	1	1	1	1	1	1	1
sulfuric acid	5	5	5	5	5	5	5	5

Standard: 0–48μg/ml glucose (Sigma, MO, USA), CS-PS sample solution: CS-PS (25 mg) was dissolved in 250mL of deionized water and centrifuged at 1000 g for 10 min. The CS-PS solution (2 mL) was added into the testing tube for detection.

The gypenosides were analyzed by high-performance liquid chromatography (HPLC) (Agilent 1200 Series, Santa Clara, CA, USA) [4], [5]. The gypenosides contain 10.34% gypenosides XLIX and 16.58% gypenosides A (Fig. 5).
